# Comprehensive Characterization of *Mycoplasmosis bovis* ST52 Strain 16M Reveals Its Pathogenicity and Potential Value in Vaccine Development

**DOI:** 10.3390/vetsci12111044

**Published:** 2025-11-01

**Authors:** Liang Zhang, Tingwei Wang, Jilong Wang, Yunfei Zhang, Tianyu Zhang, Zhiyong Wu, Wenhui Wang, Hongjun Yang

**Affiliations:** 1 College of Veterinary Medicine, Gansu Agricultural University, Lanzhou 730070, China; moon334401@163.com (L.Z.);; 2 Shandong Key Laboratory of Animal Disease Control and Breeding, Institute of Animal Science and Veterinary Medicine, Shandong Academy of Agricultural Sciences, Jinan 250100, China; 3 Key Laboratory of Livestock and Poultry Multi-omics of MARA, Jinan 250100, China

**Keywords:** *Mycoplasmosis bovis*, drug resistance, pathogenicity, immunogenicity, genetic stability

## Abstract

**Simple Summary:**

*Mycoplasmosis bovis* is a pathogen that causes respiratory diseases and other infections in cattle, leading to significant economic losses in the farming industry. This study aimed to evaluate a specific strain called 16M, isolated from a Chinese outbreak, to determine its suitability for use in vaccine testing and development. Researchers conducted laboratory tests and calf experiments, revealing that the 16M strain grows robustly, shows resistance to certain antibiotics, and causes severe lung damage in infected animals. However, when inactivated and used as a vaccine, it effectively stimulated immune protection in calves, reducing disease severity after exposure. The strain also maintained stable characteristics over multiple generations. This work provides a foundation for developing effective vaccines against *Mycoplasma bovis*, which could help control outbreaks, improve cattle health, and support sustainable livestock production.

**Abstract:**

This study aimed to characterize *Mycoplasmosis bovis* strain 16M—a highly virulent isolate from one Chinese outbreak—as a candidate for challenge models and inactivated vaccine development. We assessed strain 16M through morphological observation, PCR identification, drug susceptibility testing, growth titer and biofilm quantification, immunological profiling, and calf challenge experiments. We used genomic resequencing to evaluate the genetic stability across 150 passages. Classified as the prevalent ST52 lineage in China, strain 16M showed phylogenetic proximity to strain 08M and exhibited multidrug resistance (notably to macrolides). It achieved higher titers and stronger biofilm formation than other isolates and the reference strain PG45. In calves, intratracheal inoculation with 16M induced universal infection, severe pulmonary consolidation, and peribronchial cuffing, with significantly higher disease scores (*p* < 0.01). The inactivated 16M vaccine elicited elevated antigen-specific IgG titers, PBMC proliferation, and IFN-γ production versus PG45. Post challenge, immunized calves showed reduced pathological lesions, shorter bacterial shedding, and lower disease scores than the infected controls (*p* < 0.05). Genetic stability was confirmed for virulence-associated genes (e.g., adhesion proteins), with stable titers and biofilm production within 50 generations. Strain 16M combines high virulence for challenge modeling and industrial-scale vaccine suitability, owing to its robust growth, stable immunogenicity, and genetic consistency.

## 1. Introduction

*Mycoplasmosis bovis* (*M. bovis*), belonging to the family *Mycoplasmataceae* and order *Mycoplasmatales*, is the smallest known independent living prokaryotic organism [1]. Lacking a cell wall, it is highly sensitive to environmental factors but resistant to antibiotics that target bacterial cell wall synthesis, such as beta-lactams [2]. With a relatively small genome size of around 1080 kilobase pairs and a G+C content of 27.8% to 32.9% [3], its limited genetic capacity restricts its biosynthetic and metabolic capabilities. *M. bovis* is a significant pathogen in the cattle industry [4]. Since its first isolation from a bovine mastitis case in the US in 1961 [5], it has become a global concern. This pathogen causes various diseases in cattle, including mastitis [6], pneumonia [7], arthritis [8], otitis media [9], and reproductive diseases [10,11,12], leading to substantial economic losses. Studies show that *M. bovis* has a high prevalence worldwide [13,14,15]. In New Zealand, the *M. bovis* outbreak, which was first detected in 2017, affected over 1800 farms and led to the culling of nearly 160,000 cattle, costing NZ$203 million by 2020 [16]. In China, since the first report of *M. bovis* infection in 1983 [17], the prevalence of this pathogen has become increasingly severe. In particular, after the first report of *M. bovis* pneumonia in Hubei Province in 2008 [18], *M. bovis* has occurred in multiple regions [19] and poses a potential risk of vertical transmission [20].

As a globally distributed pathogen, *M. bovis* poses a serious threat to the cattle industry and requires extensive attention and effective prevention. Currently, *M. bovis* infection control relies on antibiotics [16] such as macrolides, aminoglycosides, and quinolones. However, increasing antibiotic resistance due to frequent and improper use has become a major concern [21]. Culling and eliminating *M. bovis*-positive cattle are primary control measures but need laboratory support and significant funding [16]. Consequently, there is an urgent need for effective commercial vaccines. The available inactivated vaccine in the US lacks proven efficacy, mainly due to low protection rates and inability to prevent pathogen transmission [22,23]. While the attenuated vaccine shows promise in protecting calves [24], it carries risks of virulence reversion and pathogenicity. A clinically proven and commercially available *M. bovis* vaccine remains unavailable worldwide [16]. This is primarily due to the lack of well-defined protective antigens and protective immune response types [25]. In addition, the development of such vaccines has been significantly constrained by challenges in obtaining high-titer strains and establishing stable animal models. One promising inactivated vaccine candidate strain depends more on strain characteristics, including prevalence, culture titer, immunogenicity, and genetic stability. At present, MLST is generally used for *M. bovis* serotyping internationally [26]. The higher titer in in vitro culture will reduce the cost of vaccine production, and more stable heritability facilitates vaccine production and product stability. In addition, highly virulent strains are essential for the development of *M. bovis* vaccines, as they constitute a prerequisite for establishing a stable challenge model [4]. The immunogenicity is primarily evaluated from the perspectives of humoral immunity and cellular immunity. The former mainly involves the antibody levels induced by antigens, while the latter primarily includes lymphocyte proliferation and cytokine production [27]. Moreover, biofilm of *Mycoplasma* is not only associated with environmental resistance and immunogenicity but also positively correlates with the virulence [28], suggesting that biofilm-based testing as a reference factor can be used for the preliminary selection of highly virulent *M. bovis* strains. Therefore, this study aimed to comprehensively characterize a prevalent and virulent *Mycoplasmopsis bovis* ST52 strain, 16M, by evaluating its biological characteristics, genomic features, pathogenicity, and immunogenicity, to assess its potential as a challenge strain and an inactivated vaccine candidate.

## 2. Materials and Methods

### 2.1. Strain Source

The reference strain PG45 (ATCC25523) was purchased by the China Institute for Veterinary Drug Control, Beijing, China. *M. bovis* strain 16M was isolated from a dairy farm in Shandong Province, China, during a 2016 *M. bovis* infection outbreak. During this epidemic, the *M. bovis* infection rate and mortality rates in calves (under 4 months old) were 84.35% (194/230) and 35.05% (68/194), respectively, and the infection rate in corresponding lactating cows was 21.30% (49/230). These clinical data showed the strong infectivity and pathogenicity of 16M. After isolation, culture, and three generations of cloning and purification, it was deposited at the China Center for Type Culture Collection (CCTCC M2019235). Other *M. bovis* isolates in this study were lyophilized and preserved in our lab.

### 2.2. Isolation and Culture

The methods of isolation and culture for *M. bovis* are referenced in previous research [29], but some modifications are described below. The modified *M. bovis* broth liquid medium was used to isolate and culture *M. bovis*, including 21 g/L pleuropneumonia like organism (PPLO) broth medium and yeast extract (BD Biosciences, San Jose, CA, USA), 10% DMEM (Dulbecco’s Modified Eagle Medium) and 20% horse serum (Thermo Fisher Scientific, Waltham, MA, USA), 45 μg/mL phenol red, 0.01 mg/mL salmon sperm DNA and 300 U/mL penicillin (Solarbio, Beijing, China), and 0.2% pyruvate (Haibo, Qingdao, China). The solid medium, which was based on liquid media supplemented with 2% agar, was used for isolation, purification, and counting. The pH of the two culture media was adjusted to 7.8. For *M. bovis* culture, the passages and scale-up of *M. bovis* were performed at a 1:5 ratio and at a 1:10 ratio, respectively. The strains used in the experiment were harvested during the logarithmic growth period. For the cloning and purification of *M. bovis*, the *M. bovis* suspension was streaked onto solid medium plates and incubated for 5 days at 37 °C in an atmosphere of 10% CO_2_.

### 2.3. Identification of M. bovis

The colony characteristics of *M. bovis* were observed under an optical microscope after a 5-day culture period. For further morphological observation, we aliquoted 20 μL of *M. bovis* suspensions and fixed them onto copper grids with carbon film for 3–5 min. Filter papers were used to absorb the excess liquid. Subsequently, the copper grids were stained using 2% phosphotungstic acid for 1–2 min and blot-dried using filter papers. The copper grids were observed under a transmission electron microscope (TEM: JEM-2100, JEOL Ltd., Tokyo, Japan), and the images were recorded. The culture titer of the 16M strain was measured in colony-forming units (CFU) by a fold ratio dilution and plate culture. Then, 5 mL of the bacterial suspension was centrifuged at 12,000 rpm for 15 min and used for DNA extraction. DNA was extracted using a SteadyPure Bacterial Genomic DNA Extraction Kit (Accurate Biology, Changsha, China) and stored at −20 °C until PCR testing. Specific PCR (using primers targeting the uvrC gene [30]) and biochemical tests were used for the identification and detection of *M. bovis* in nose swabs, following the previous research [31].

### 2.4. Drug Resistance Test

The minimal inhibitory concentration (MIC) assay was used to assess the antimicrobial susceptibility of the 16M strain [32,33]. The *M. bovis* suspension was diluted to approximately 10^5^ color-changing units (CCU) per mL, filtered, and then added to the wells of a 96-well microplate with the antimicrobial agents. The final test dilutions in the total volume of 200 μL per well were 1–1024 μg/mL for tylosin (TYL), tilmicosin (TIM), gamethromycin (GAM), tildipirosin (TIL), tylvalosin (TYV), erythromycin (ERY), tulathromycin (TUL), terramycin (TER), tetracycline (TET), doxycycline (DEO), enrofloxacin (ENR), ciprofloxacin (CIP), marbofloxacin (MAR), spectinomycin (SPE), kanamycin (KAN), lincomycin (LIN), and florfenicol (FLO) and 0.001–1 μg/mL for tiamulin (TIA). The medium containing *M. bovis* and the medium alone were used as the negative and blank controls, respectively, for each plate. The MIC values of the *M. bovis* PG45 strain were determined for quality control and compared to previously published values [34]. The above experiments were independently repeated three times to ensure the reliability and repeatability of the data.

### 2.5. Biofilm Detection

The biofilm formation ability of *M. bovis* clinical isolates was assessed using reported methods [24,35]. The titer of fresh cultures of clinical isolates was adjusted to 10^8^ CFU/mL and diluted 10-fold with liquid medium. Next, 200 μL was added to each well of a 96-well microplate, with three replicates per isolate. The liquid medium without *M. bovis* was used as the negative control. After sealing the plate with a cover and placing it in a 37 °C incubator for 72 h, the formation of biofilms was assessed. The culture plate was gently washed three times with 250 µL of sterile PBS to remove free-floating *M. bovis*. The plate was then placed in a 65 °C constant temperature drying oven for 30 min to fix the sample, stained with 1% crystal violet (250 μL/well) for 5 min, washed three times with phosphate salt buffer (PBS), and air-dried at room temperature. Subsequently, the samples were dissolved in 250 μL of 95% ethanol for 10 min until the crystal violet was completely dissolved. Then, 200 µL of the eluted solution was transferred into a new 96-well microplate for detection, and the absorbance of the eluate was measured at 570 nm (OD570 value) using a microplate reader (BioTek, Winooski, VT, USA).

### 2.6. Pathogenicity and Challenge Study

To verify the virulence of the isolated *M. bovis* 16M strain, fifteen one-month-old, clinically healthy dairy calves were selected from a commercial farm with no history of *M. bovis* infection. Prior to the study, all calves were confirmed negative for *M. bovis* via weekly serological testing (iELISA, IDEXX Laboratories, Westbrook, ME, USA) and PCR detection of nasopharyngeal swabs over a two-week observation period. The calves were randomly allocated into three groups of equal size (*n* = 5) by a random number generator: the 16M challenge group (10^10^ CFU/mL), the PG45 challenge group (10^10^ CFU/mL), and the negative control (PBS) group. All groups were challenged through tracheal injection with 4 mL. The three groups were segregated in different spaces and observed for 36 days. Serum samples were collected weekly for antibody titer via iELISA, and nasopharyngeal swabs were examined weekly using PCR. Drawing on the experience of the previous pre-experiment, we added the quantitative indicators of clinical signs, the bacterial discharge time, and the microscopic pathology based on the pulmonary gross score. The total score was 20 points, and the higher the score, the higher the degree of incidence. The specific scoring criteria are shown in Table S1. Changes in appearance (rough coat) or behavior were observed and recorded. After the scheduled study completion date, all calves were euthanized by intravenous injection of sodium pentobarbitone, and the gross lesions were examined. In addition, bacteria were recovered from lung samples for identification via PCR. Tissues were collected for histopathological observation. The lung tissue was cut (about 1 cm^3^) and fixed immediately with 4% neutral buffered paraformaldehyde and sent to Wuhan Servicebio Technology Co., Ltd. (Wuhan, China) to produce consecutive tissue sections for hematoxylin–eosin staining. All clinical observations and pathological scoring were performed by three veterinarians blinded to the group assignments of the calves to avoid bias. A cumulative clinical score was calculated for each animal daily. In order to test the effect of the 16M strain as a vaccine strain for protection against virulence, the vaccinated group and NC groups were challenged via tracheal injection with 4 mL (10^10^ CFU/mL) of virulent *M. bovis* field strain 16M on day 35 post-vaccination. Clinical monitoring and pathological scoring followed the protocol described.

### 2.7. Growth Inhibition Experiment

To test the inhibitory effect of the hyperimmune serum from *M. bovis* strain 16M on different dissociation strains, the rabbit antiserum was raised against the *M. bovis* 16M strain, referring to the previous reports [36,37]. The growth inhibition test was conducted according to the method described in the literature [38,39], with the criterion for effective growth inhibition being an inhibition zone diameter greater than 2 mm. In addition to PG45 and 16M, another 47 isolates stored in our lab were used for this test, which were isolated from dairy herds in China. The test was performed in triplicate. The diameters of the inhibition zones were measured and compared using a one-way ANOVA followed by Tukey’s post hoc test.

### 2.8. Vaccine Preparation

After titer determination, the fresh *M. bovis* 16M strain and PG45 standard strain cultures were centrifuged at 12,000 rpm for 10 min to collect the bacterial pellets. The pellets were resuspended in 15 mL PBS of 0.4% formaldehyde per tube and incubated at 37 °C for 24 h to inactivate. After inactivation, 50% sterile sodium thiosulfate solution was added to achieve a final concentration of 1.5%, and the mixture was incubated at 37 °C for 1 h to neutralize and terminate the inactivation. The suspension was then adjusted to 4.0 × 10^10^ CFU/mL with PBS and retained. The 206 adjuvant (SEPPIC, Puteaux, France) and suspension were mixed in a 54:46 ratio, emulsified on a shaker at 120 rpm for 1 h at 30 ± 1 °C, to produce the inactivated *M. bovis* vaccine, which was stored in a refrigerator at 4 °C. The vaccine was plated onto blood agar and Eaton’s medium to screen for bacteria and confirm *M. bovis* inactivation, respectively, and three sequential subcultures at 3–4-day intervals remained sterile.

### 2.9. Evaluation of the Immune Effect

Fifteen 2-month-old calves (negative for antibody via iELISA and *M. bovis* using PCR) were randomly divided into a 16M-vaccinated group, a PG45-vaccinated group as the positive control, and a negative control (NC), with 5 calves in each group. Via muscle injection, the 16M-vaccinated group received 2 mL of the inactivated 16M strain vaccine, the PG45-vaccinated group received 2 mL of the inactivated PG45 strain vaccine, and the NC group was administered 2 mL of normal saline. Serum samples were collected from all calves before immunization (day 0) and post-immunization on days 7, 14, 21, 28, and 35. Anti-*M. bovis* IgG antibodies were measured using a commercial ELISA kit (*MYCOPLASMA BOVIS* ELISA KIT, BIO K 302, Bio-X, Belgium) according to the manufacturer’s protocol.

On day 35 post-vaccination, anticoagulant blood from the experimental, positive, and negative groups was aseptically collected to isolate bovine peripheral blood mononuclear cells (PBMC) for the cell immune effect evaluation, referring to the relevant study [40]. In 96-well cell culture plates, 100 μL of freshly isolated PBMC (3000 cells/well) were added to each well, along with 100 μL of heat-inactivated *M. bovis* 16M strain (MOI = 1:100), Concanavalin A, or 1640 culture medium (with 10% FBS). Each treatment was set with three replicates. After incubation in a 37 °C, 5% CO_2_ atmosphere for 46 h, 12 μL of CCK-8 solution was added to each well, followed by further incubation for 2 h. Absorbance was measured at 450 nm and 630 nm (reference wavelength), with the absorbance value at the reference wavelength deducted. The concentration of IFN-γ in the cell culture supernatants from all groups was measured using a commercial ELISA kit (Area, TB-IFN.K005, Qingdao, China), following the manufacturer’s instructions.

### 2.10. Genome Sequencing and Comprehensive Analysis

The fresh suspension of *M. bovis* (50 mL) was centrifuged at 12,000 rpm for 15 min. The bacterial precipitation was heated for 30 min at 65 °Cand sent in dry ice for de novo genome sequencing to Beijing Novogene Bioinformatics Technology Co., Ltd. (Novogene, Beijing, China). Pathogen–host Interactions (PHI) [41], Virulence Factors of Pathogenic Bacteria (VFDB) [42], the Antibiotic Resistance Genes Database (ARDB) [43], and the Comprehensive Antibiotic Research Database (CARD) [44] were used to perform the above analyses. Carbohydrate-active enzymes were predicted using the Carbohydrate-Active enZYmes Database [45].

Comparative genomics analysis included the genomic synteny, single-nucleotide polymorphism (SNP), insertion and deletion (indel), structural variation (SV) annotation, genomic alignment, and genome visualization, which were performed using MUMmer 3.0 [46] and LASTZ 1.04.03 [47] tools. Genomic synteny was analyzed based on the results of the alignment. A phylogenetic tree was constructed using the NCBI genome phylogenetic tree. SNPs, indels, and SVs were identified through genomic alignment results among samples, utilizing MUMmer 3.0 and LASTZ 1.04.03. To depict the annotation information, a genome overview was created using Circos. ST typing was performed on the PubMLST data [26].

### 2.11. Genetic Stability Testing

To determine the optimal passage number for the production strain of *M. bovis* 16M, the biological characteristics of different passages were compared. The 16M strain was continuously passaged, and the culture titers of the 50th, 100th, and 150th passages were measured. Bacterial genomic DNA was extracted from these passages using a kit (Accurate Biology, Changsha, China) and sent to Hangzhou Linkcare Bio-Pharma Technology Co., Ltd. (Linkcare, Hangzhou, China)for resequencing to assess the genetic variation.

### 2.12. Statistics and Analysis

All data were organized in Excel and analyzed statistically using GraphPad Prism 8.0.2. The Shapiro–Wilk test was used to assess the normality of the data distribution for all relevant datasets prior to selecting parametric or non-parametric tests (*p* > 0.1). For normally distributed data, comparisons between two groups were performed using the unpaired Student’s *t*-test, while comparisons across more than two groups were analyzed by one-way ANOVA followed by Tukey’s post hoc test for multiple comparisons. All data were presented as mean ± standard deviation (SD). The significance levels *p*-value < 0.05 (*), *p*-value < 0.01 (**), *p*-value < 0.001 (***), and *p*-value < 0.0001 (****) indicated significant differences, and *p*-value > 0.05 means no significant difference (ns).

## 3. Results

### 3.1. Molecular and Phenotypic Characteristics of M. bovis Strain 16M

**Figure 1:**
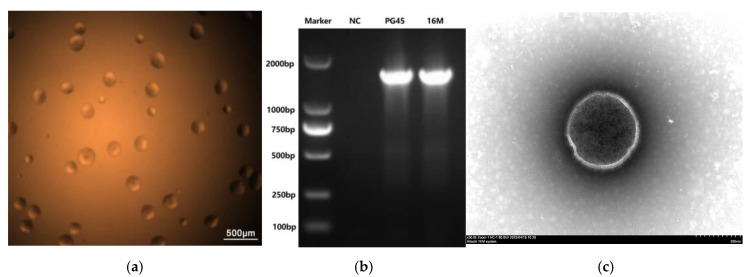
The morphology and PCR identification of the *M. bovis* 16M strain. (**a**) The colonial morphology of the *M. bovis* 16M strain under an optical microscope (40×); (**b**) the identification of the *M. bovis* strain 16M using specific PCR; (**c**) the mycelial morphology of the *M. bovis* 16M strain observed under TEM.

The colonies of *M. bovis* strain 16M were smooth-edged, with a centrally raised elevation, and ranged in size from 100 μm to 200 μm when cultured on solid medium (Figure 1a). Specific PCR identification revealed the 16M strain as *M. bovis* (Figure 1b). Highly variable size and morphology were observed in the morphological observation using TEM (Figure 1c). A single copy of isolated *M. bovis* was expanded, lyophilized, and kept at −20 °C in the China Center for Type Culture Collection (CCTCC M2019235).

**Figure 2:**
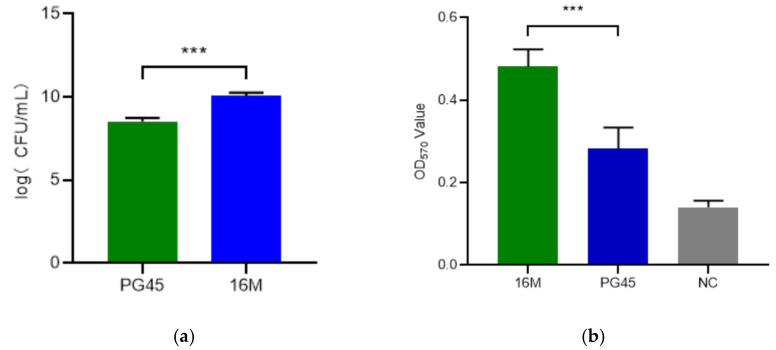
The titers and biofilm of strain 16M and strain PG45. (**a**) The titers of strain 16M and strain PG45; (**b**) The biofilm of strain 16M and strain PG45. *** means *p* < 0.001.

The culture titer of strain 16M was determined as 2 × 10^10^ CFU/mL (Figure 2a), significantly higher than PG45 (*p* < 0.001). Additionally, the biofilm formation assay value of the 16M strain was significantly higher compared to the PG45 and NC, with a *p*-value < 0.001, indicating that the 16M strain may exhibit more abundant membrane proteins and greater virulence potential (Figure 2b).

### 3.2. Drug Susceptibility Analysis

**Figure 3:**
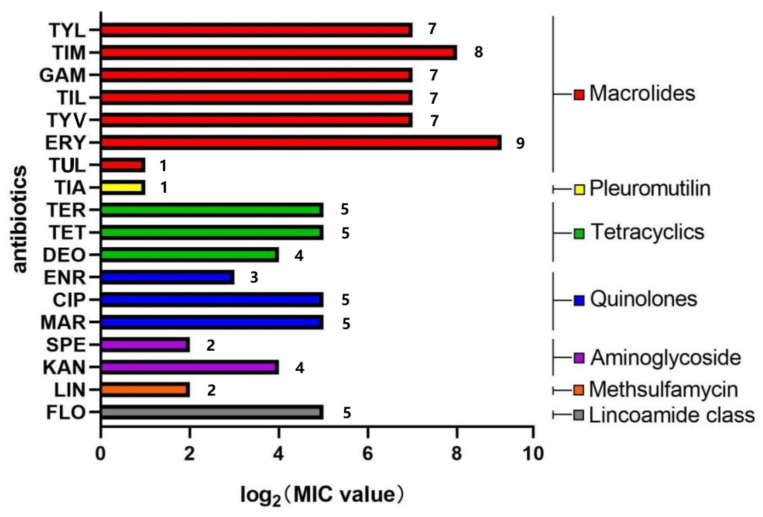
Results of the drug resistance analysis on the *M. bovis* strain 16M.

According to the previous study [34], the antimicrobial susceptibility of the *M. bovis* 16M strain was determined. It was sensitive to TUL (2 μg/mL), TIA (2 μg/mL), ENR (8 μg/mL), SPE (4 μg/ml) and LIN (4 μg/mL), and intermediate to TER (32 μg/mL), TET (32 μg/mL), DEO (16 μg/mL), CIP (32 μg/mL), MAR (32 μg/mL), KAN (16 μg/mL) and FLO (32 μg/mL), and resistance to TYL (128 μg/mL), TIM (256 μg/mL), GAM (128 μg/mL), TIL (128 μg/mL), TYV (128 μg/mL) and ERY (512 μg/mL). The specific drug resistance data for the 16M strain were shown in Figure 3. The 16M strain showed higher resistance to macrolides than to other drugs used in the study.

### 3.3. Immunological Estimate of M. bovis Strain 16M

**Figure 4:**
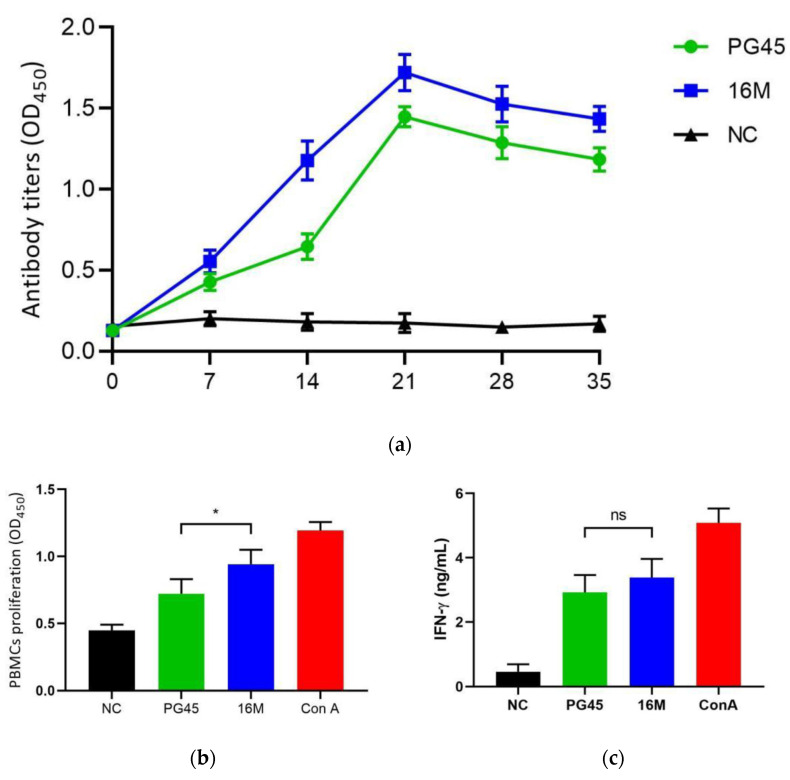
Comparison of humoral and cellular immune responses between the PG45 and 16M strains. (**a**) The antibody levels over time following vaccination between the PG45 group and the 16M group; (**b**) the contrast of PBMCs proliferation levels between the PG45 group and the 16M group; (**c**) the contrast of IFN-γ levels between the PG45 group and the 16M group. * means *p* < 0.05, ns means *p* > 0.05.

The growth inhibition assay demonstrated that the rabbit antiserum raised against the *M. bovis* 16M strain effectively suppressed the proliferation of PG45, 16M, and the other 47 isolates (zones of inhibition of >2 mm). In the calf immunization trial, the 16M strain exhibited superior immunogenicity compared to PG45, inducing higher titers of *M. bovis*-specific antibodies (Figure 4a). Notably, the antibody levels in the 16M group surpassed those of the PG45 group at critical timepoints, with statistically significant differences observed on days 14 and 21 post-immunization (*p* < 0.01). Statistical analysis of the PBMC proliferation in the 16M-vaccinated group and PG45-vaccinated group demonstrated that both vaccines induced antigen-specific lymphocyte proliferation upon stimulation with inactivated *M. bovis* antigen. The experimental groups exhibited significantly higher proliferation responses compared to the negative control (*p* < 0.001). Notably, the 16M-vaccinated group, as shown in Figure 4b, had superior proliferation activity relative to the PG45-vaccinated group, with a statistically significant difference between the two vaccine formulations (*p* < 0.05). For IFN-γ secretion analysis, both vaccines elicited detectable IFN-γ production in antigen-stimulated PBMCs, and the experimental groups again displayed significantly elevated IFN-γ levels compared to the negative control (*p* < 0.05). However, while the 16M-vaccinated group exhibited higher IFN-γ responses than the PG45-vaccinated group, this difference did not reach statistical significance (*p* > 0.05), as shown in Figure 4c. The above results indicated that the inactivated 16M strain can induce higher levels of humoral immune response and cellular immune response in the organism compared to PG45 as a standard strain of *M. bovis*.

### 3.4. Pathogenicity of the M. bovis 16M Strain

**Figure 5:**
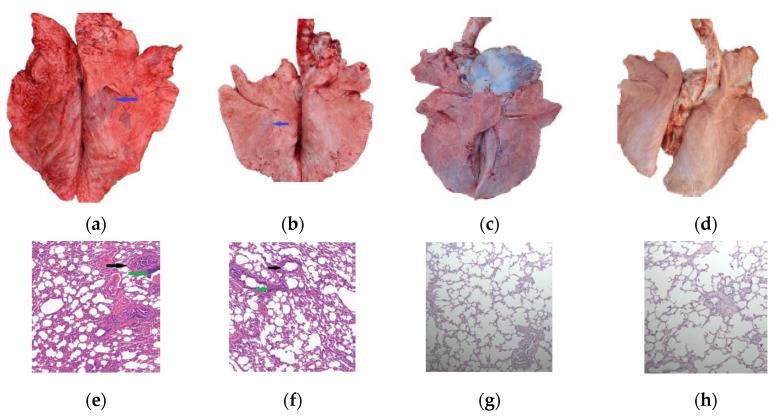
The gross lesions and pathological changes in the challenge test. (**a**) The gross lesions in the 16M-infected group; (**b**) the gross lesions of PG45-infected group; (**c**) the gross lesions in the 16M-vaccinated group; (**d**) the gross lesions in the NC group; (**e**) the pathological changes in the 16M-infected group (100×); (**f**) the pathological changes of PG45-infected group (100×); (**g**) the pathological changes in the 16M-vaccinated group (100×); (**h**) the pathological changes in the NC group (100×).

Within 10 days post-challenge, calves in both the PG45 and 16M groups exhibited varying degrees of elevated body temperature. However, the mean body temperature of these groups showed no significant difference compared to the control group after this initial period. Clinical observations revealed that the experimental calves presented with respiratory symptoms of differing severity levels, occasionally accompanied by conjunctivitis. No cases of arthritis were observed in any of the challenged animals during the study period. The gross lesions and pathological changes of the 16M group were compared with the PG45 group and the NC groups, as shown in Figure 5. The pulmonary consolidation areas (indicated by blue arrows in Figure 5a,b) were significantly more prevalent in the 16M strain group compared to the PG45 group. Notably, the 16M strain group exhibited more pronounced peribronchial cuffing structures (marked by black arrows in Figure 5e,f) and inflammatory cell aggregation (highlighted by green arrows in Figure 5e,f). These pathological features indicate that the pathogenicity of the 16M strain in the challenge model was higher than that of the PG45 strain with the same challenge dose of 4 × 10^10^ CFU per calf.

**Figure 6:**
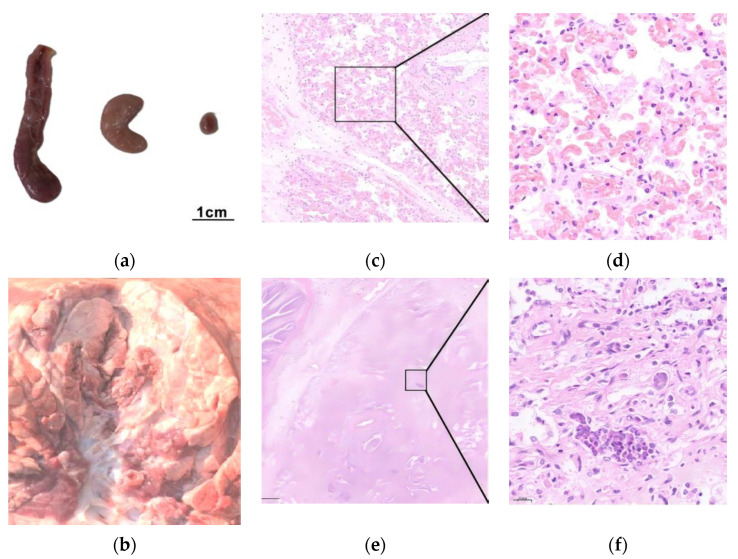
Representative necropsy photographs and histopathological sections from the 16M group. (**a**) hilar lymph nodes: the left is the 16M group, the mid is the PG45 group, and the right is the NC group (normal); (**b**) lung cross section; (**c**) significant interstitial thickening (100×); (**d**) enlarged view of the selected area in figure (**c**) (400×); (**e**) peribronchovascular cuffing in consolidated areas (100×); (**f**) enlarged view of the selected area in figure (**e**) (400×).

Representative necropsy photographs and histopathological sections from the 16M group are presented in Figure 6. The principal pathological findings included the following: marked enlargement of hilar lymph nodes (>5 cm), as shown in Figure 6a; multifocal pulmonary consolidation with sharply demarcated boundaries between lesional and normal parenchyma, and bronchi and bronchioles contained white-to-pale yellow mucopurulent exudates (Figure 6b); significant interstitial thickening in affected pulmonary regions (Figure 6c); peribronchovascular cuffing in consolidated areas, characterized by inflammatory cell infiltration surrounding bronchioles and blood vessels (Figure 6e); alveolar structural alterations, including collapse or obliteration of alveolar spaces, thickened septa with sparse mononuclear cell infiltration, and focal hemorrhage (Figure 6d,f).

### 3.5. Challenge Model and Vaccine Protection

Based on the disease scoring criteria, the overall scores for the PG45, 16M, and NC groups were calculated from the clinical signs, bacterial shedding duration, gross lesions, and pathological changes, as shown in Table 1. The statistical analysis revealed that the PG45 group (mean 8.6 ± 1.67) and the 16M group (mean 13.6 ± 2.70) had significantly higher scores than the NC group (*p* < 0.0001). Moreover, the 16M group had significantly higher scores than the PG45 group (*p* < 0.05). The challenge model using 16M as the attack strain has higher stability and resolution under the new evaluation criteria.

Post challenge, although the gross and histopathological alterations persisted in the 16M-vaccinated calves (Figure 5c,g), these lesions were significantly reduced compared to those observed in the 16M group. In terms of the overall disease score, the 16M-vaccinated calves exhibited significantly lower scores (4.0 ± 1.41) compared to unvaccinated calves (13.6 ± 2.70; *p* < 0.001). The clinical symptom severity was markedly reduced in the 16M-vaccinated group (0.6 ± 0.55) relative to 16M-infected controls (2.6 ± 1.14; *p* < 0.05), while the duration of bacterial shedding shortened substantially in the 16M-vaccinated calves (0.8 ± 0.45 days) versus unvaccinated counterparts (3.2 ± 0.9; *p* < 0.01). Furthermore, gross lesion scores demonstrated a significant reduction in the 16M-vaccinated calves (1.2 ± 0.45) compared to unvaccinated calves (3.6 ± 0.89; *p* < 0.01); similarly, histopathological analysis revealed attenuated pathology in the 16M-vaccinated calves (1.4 ± 0.55) relative to unvaccinated calves (4.2 ± 0.84; *p* < 0.001) after challenge. These results demonstrated that the inactivated 16M strain at a dosage of 4.0 × 10^10^ CFU per dose conferred significant protection following a two-dose immunization regimen, particularly in reducing the duration of bacterial shedding and the severity of clinical symptoms.

### 3.6. Comparative and Comprehensive Genome-Wide Analysis

**Figure 7:**
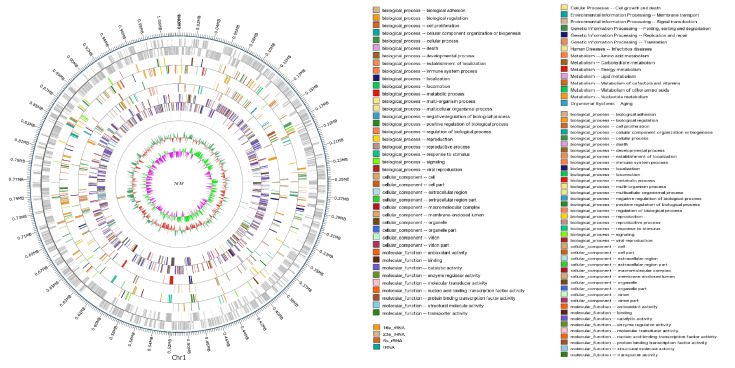
Chromosome atlas of the *Mycoplasmosis bovis* strain 16M.

The genome of the *M. bovis* strain 16M comprises a single circular chromosome of 1,019,748 bp with a GC content of 29.24%. A total of 885 protein-coding genes were annotated, representing 72.32% of the genome, with 754 genes confirmed as functional coding sequences. Functional categorization via COG (Clusters of Orthologous Groups) revealed 284 genes (32.09%) distributed across 21 functional categories, including 83 genes (29.23%) associated with metabolic pathways such as amino acid transport and energy metabolism. KEGG pathway analysis further identified 157 genes involved in metabolic processes, 101 genes in genetic information processing (e.g., replication, transcription), 31 genes in environmental signal transduction, and 14 genes regulating cellular processes (e.g., cell motility). Notably, intergenic regions exhibited a lower GC content (27.68%) compared to coding regions (29.9%), reflecting evolutionary constraints in non-coding sequences. The genome harbors 41 non-coding RNAs, including 34 tRNAs, 4 rRNAs (2 × 5S rRNA, 1 × 16S rRNA, 1 × 23S rRNA), and 3 ncRNAs, alongside 60 tandem repeats and 92 dispersed nuclear elements, suggesting roles in genomic plasticity and adaptation. Interestingly, strain 16M belonged to ST52, which is absolutely predominant among Chinese isolates (42/48) according to the pubMLST database in Figure S1. The complete genome sequence was deposited in GenBank under accession number CP038861.1, and the detailed genomic architecture and functional annotations are illustrated in Figure 7. This genomic profile provides critical insights into the pathogenicity mechanisms and metabolic adaptations of strain 16M, positioning it as a key reference for comparative studies with other *M. bovis* strains.

**Figure 8:**
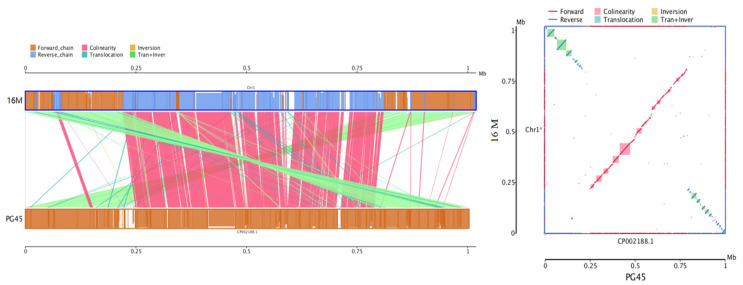
Collinearity analysis of the complete genome between *M. bovis* strains 16M and PG45.

The 16M genome size was 16,344 bp longer than the PG45 genome, and the overall sequence similarity of 16M and PG45 was calculated to be 98.66%. The genome collinearity analysis between 16M and PG45 revealed that their genomic structures do not exhibit a high degree of synteny, with only 220 blocks containing 269 genes in the comparison (Figure 8). Approximately 38.27 kb translocation and inversion involving 116 genes were found at both ends of the genome, and 49.64 kb collinearity of 81 genes was mainly distributed in the middle of the genome. A 30.11 kb translocation of 68 genes and a 5.4 kb inversion of four genes interspersed the genome. The number of SNPs in the genome of 16M was 12,098 bp against the PG45 strain, including 6386 synonymous SNPs, 3853 nonsynonymous SNPs, and 1800 intergenic SNPs. The numbers of SNPs, leading to start nonsynonymous mutation, stop nonsynonymous mutation, and premature nonsynonymous mutation, were 2, 1, and 62, respectively. The premature nonsynonymous mutation was caused by more pseudogenes in the 16M strain than in the PG45 strain. The phylogenetic tree was built as a circle tree based on the NCBI genome database of 72 *Mycoplasma* strains using the neighbor-joining method (Figure S1). According to this phylogenetic tree, the *M. bovis* 16M strain and the PG45 strain were classified into different evolutionary branches and have the closest relationship with strain 08M.

All the identified virulence genes were found in the *M. bovis* 16M strain and are shown in Table S2. In addition, 17 potential virulence genes were identified through Diamond soft search against the VFDB database. Furthermore, 29 potential virulence genes were predicted by the PHI database. Three potential drug-resistant genes conferring resistance to fluoroquinolones, rifampicin, and aminocoumarin were found by CARD. Six genes were identified twice in three databases, including gyrA, which confers resistance to fluoroquinolones, lipoate protein ligase A1, pyruvate dehydrogenase pdhB, UTP-glucose-1-phosphate uridylyltransferase, DNA gyrase subunit A, and magnesium ion transporter. Further, 44 genes were predicted to be involved in putative lipoprotein and lipoprotein metabolism of *M. bovis* strain 16M (Table S3). Interestingly, the gene cluster encoding the VSPs in the genome of the *M. bovis* 16M strain consisted of 30 open reading frames (22 encode VSPs), which was more than PG45 (15 open reading frames and 13 encoding VSPs).

### 3.7. Genetic Stability

Whole-genome resequencing analysis was performed on the *M. bovis* strain 16M at passages 50, 100, and 150, using the genomic sequence of the high-passage 16M strain as the reference (Table 2). The comparative analysis revealed no significant differences (*p* > 0.05) in the culture titer or biofilm formation between the high-passage strain and its subcultures up to passage 50.

## 4. Discussion

In the currently reported commercial inactivated *M. bovis* vaccines, the antigen content ranges from 3.44 × 10^8^ to 3 × 10^9^ CFU per dose [48,49,50], significantly lower than other bacterial inactivated vaccines, such as the Pasteurella inactivated vaccine. Notably, multiple in vitro studies have demonstrated that *M. bovis* elicits substantial immune responses only at a multiplicity of infection (MOI) ≥ 1,00 [50,51]. This discrepancy suggests that the insufficient antigen load in existing vaccines may partially account for their suboptimal protective efficacy. Conventional culture methods for *M. bovis* generally yield low titers, rendering commercial vaccine production economically unfeasible when attempting to increase the antigen concentration under the current cost and market constraints [52]. In this study, strain 16M cultured in optimized medium achieved a high titer of 2 × 10^10^ CFU/mL, demonstrating the potential to resolve these technical bottlenecks through enhanced culturing efficiency.

Clinically, *M. bovis* infections in calves are frequently associated with co-pathogens, leading to severe clinical manifestations and high mortality rates [3,53]. In contrast, single-agent infections typically induce milder pathological changes and subacute clinical progression with low mortality [24,48,49]. The controlled experimental environment ensured pathogen-specific exposure, resulting in localized gross lesions smaller than those observed in natural polymicrobial infections. Intrabronchial inoculation directed lesion progression along the bronchial tree toward the lung periphery (Figure 6b), whereas the gross lesion evaluation primarily quantified surface-visible pathology, necessitating complementary histopathological evaluation to capture full disease severity. Furthermore, effective vaccines should not only protect susceptible hosts but also reduce bacterial shedding to mitigate transmission risks [54]. Thus, the bacterial shedding duration serves as one of the critical parameters for evaluating *M. bovis* virulence and vaccine efficacy. Compared to similar studies, the modified gross lesion scoring criteria in this study, combined with the inclusion of the bacterial shedding duration (assessed twice daily via pooled sampling) and histopathological changes as diagnostic parameters, may better reflect the dynamics of *M. bovis* infection under experimental conditions [48].

Highly virulent strains are not only a core factor in establishing infection models (ensuring pathological reproducibility and evaluation reliability) but also serve as crucial resources for deciphering virulence mechanisms and screening vaccine targets. In this study, the *M. bovis* 16M strain exhibited high virulence and robust immunogenicity, demonstrating the potential to address critical gaps in the commercial development and application of current *M. bovis* inactivated vaccines. According to the clinical data and challenge test, the high virulence of strain 16M was evidenced by high infection rates and severe pulmonary pathology in calves. Perhaps it correlates with its biofilm-forming capability—a trait linked to environmental persistence and immune evasion, which was also shown in the research on *Mycoplasma pneumoniae* [55], *Mycoplasma gallisepticum* [56], and *Mycoplasma genitalium* [57]. Chen et al. demonstrated that *M. bovis* biofilm and planktonic cells exhibit distinct immunoreactivity to bovine convalescent serum [58]. However, the vaccine efficacy of biofilm-based formulations compared to planktonic cell-derived vaccines has not yet been evaluated. The growth inhibition test is similar to virus neutralization and used for measuring growth-inhibiting antibodies [59,60]. Notably, biofilm formation and growth inhibition assays may serve as novel criteria for *M. bovis* vaccine strain screening, complementing traditional MLST analysis-based methods [61]. In addition, strain 16M outperformed the reference strain PG45 in immunogenicity, eliciting IgG titers and PMBC proliferation evidently higher post-vaccination. This disparity may stem from its unique antigenic profile and abundance, including conserved surface lipoproteins [62] and adhesion molecules [63,64], which are critical targets for protective immunity [65]. The higher IgG titers observed suggest a stronger humoral immune activation, potentially leading to better protection against *M. bovis* infection. The significant increase in antigen-specific PBMC proliferation observed in calves vaccinated with the 16M strain indicates the elicitation of a robust cellular immune response. This is crucial for protection against *M. bovis*, a pathogen known to persist intracellularly [66]. Moreover, the strong IFN-γ response and lymphocyte proliferation were observed in vaccinated calves aligned with Th1-mediated immunity, which is essential for intracellular pathogen clearance [67]. Th1-mediated immunity plays a critical role in controlling *M. bovis* infections by promoting macrophage activation, proinflammatory cytokine production (e.g., IFN-γ, TNF-α), and IgG2 antibody subclass responses, which are essential for combating the intracellular persistence and biofilm formation [67,68]. Moreover, IFN-γ enhances bacterial clearance by upregulating nitric oxide synthesis in macrophages and promoting antigen presentation [68]. Furthermore, genomic stability across 150 passages and phenotype stability across 50 passages further support its suitability for vaccine production, minimizing the risks of antigenic drift during manufacturing. The limitations include the lack of mucosal immunity assessment and longitudinal protection data beyond 35 days. Future studies should evaluate dose-dependent responses, field efficacy, and potential synergies with emerging adjuvants. Nevertheless, such advancements position the 16M strain as a superior candidate for *M. bovis* vaccine development.

In China, one attenuated vaccine and one inactivated vaccine have received administrative approval in 2025 but are not yet on the market. Therefore, no comparative trial was carried out between the vaccine prepared with the 16M strain and these commercial vaccines in this study. Additionally, the exclusive use of the 16M strain in both vaccine preparation and challenge experiments cannot confirm its universal protective efficacy, despite MLST, sequencing, and growth inhibition assays indicating that 16M belongs to a prevalent strain in China. A limitation of this study is the use of a homologous challenge model (vaccine and challenge from the same strain). While this demonstrates proof-of-concept efficacy, it may overestimate protection against heterologous field strains. Future studies should evaluate the cross-protective efficacy of a 16M-based vaccine against diverse *M. bovis* strains. With the marketing of commercial vaccines and the development of a vaccine based on strain 16M, the relevant research will be supplemented and made public in the future.

## 5. Conclusions

This study systematically investigated the biological characteristics and complete genome of the 16M strain of bacteria. It exhibits robust in vitro growth and potent in vivo pathogenicity, revealing its pathogenicity and potential value in vaccine development. In this study, the disease assessment criteria for *M. bovis* infection were optimized for the first time based on the clinical characteristics of the infection, providing a new reference for evaluating vaccine protective effects in subsequent challenge experiments. All findings facilitate further explorations of vaccines and drugs for *M. bovis* control.

## Figures and Tables

**Table 1:** The results of disease scoring for individuals in each group.

Condition	PG45 Group					16M Group					16M-Vaccinated Group					NC Group				
	1	2	3	4	5	1	2	3	4	5	1	2	3	4	5	1	2	3	4	5
Clinical signs	1	2	2	1	1	4	3	3	1	2	1	0	1	0	1	1	0	0	0	0
Shedding	2	3	3	2	2	3	4	3	2	3	2	2	2	1	2	0	0	0	0	0
Gross lesions	2	1	2	1	1	5	3	4	3	3	1	1	2	1	1	0	0	0	0	0
Histopathology	4	3	4	3	3	5	5	4	3	4	1	2	2	2	1	0	0	0	0	0
Total score	43					67					25					1				

**Table 2:** Mutation of *M. bovis* strain 16M at different generations. *** means *p* < 0.001, ns means *p* > 0.05.

Passage Number	Synonymous Variant	Missense Variant	Stop Gained	Gene Variant	Variant Rate	Biofilm (OD_590nm_)	Titers (CFU/mL)
P	0	0	0	0	0	0.481 ± 0.042	2.0 × 10^10^
F50	6	6	1	0	0.00129%	0.479 ± 0.025 ^ns^	2.0 × 10^10^
F100	5	12	2	0	0.00189%	0.389 ± 0.011 ***	2.0 × 10^10^
F150	7	21	1	6	0.00289%	0.356 ± 0.025 ***	2.0 × 10^10^

## Data Availability

The original contributions presented in this study are included in the article/Supplementary Material. Further inquiries can be directed to the corresponding author(s).

## Supplementary Materials

The following supporting information can be downloaded at: https://www.mdpi.com/article/10.3390/vetsci12111044/s1, Figure S1: MLST clustering analysis of Chinese Isolates of *Mycoplasmosis bovis*; Figure S2 Original image of Figure 1b; Table S1: The scoring criteria for challenge experiments; Table S2: The result of the prediction for potential virulence genes in the genome of *M. bovis* 16M; Table S3: Genes predicted to be involved in putative lipoprotein and lipoprotein metabolism of *M. bovis* 16M.

## Abbreviations

The following abbreviations are used in this manuscript:

*M. bovis*	*Mycoplasmosis bovis*
PPLO	Pleuropneumonia-like organism
DMEM	Dulbecco’s Modified Eagle Medium
LD	Linear dichroism
CFU	Colony-forming units
TEM	Transmission electron microscope
CCU	Color-changing units
MIC	Minimal inhibitory concentration
TYL	Tylosin
TIM	Tilmicosin
GAM	Gamethromycin
TIL	Tildipirosin
TYV	Tylvalosin
ERY	Erythromycin
TUL	Tulathromycin
TER	Terramycin
TET	Tetracycline
DEO	Doxycycline
ENR	Enrofloxacin
CIP	Ciprofloxacin
MAR	Marbofloxacin
SPE	Spectinomycin
KAN	Kanamycin
LIN	Lincomycin
FLO	Florfenicol
TIA	Tiamulin
MOI	Multiplicity of infection
ConA	Concanavalin A
PBS	Phosphate-buffered solution
ELISA	Enzyme-linked immunoadsorption assay
FBS	fetal bovine serum
PBMC	Peripheral blood mononuclear cells
IFN	Interferon
COG	Clusters of Orthologous Groups
SNP	Single-nucleotide polymorphism
SV	Structural variation
Indel	insertion and deletion
MLST	Multilocus sequence typing
PHI	Pathogen Host Interactions
VFDB	Virulence Factors of Pathogenic Bacteria
ARDB	Antibiotic Resistance Genes Database
CARD	Comprehensive Antibiotic Research Database
