# Supplementary material for: Comprehensive Characterization of Mycoplasmosis bovis ST52 Strain 16M Reveals Its Pathogenicity and Potential Value in Vaccine Development
Source: Vet Sci. 2025 Nov 1;12(11):1044. doi: 10.3390/vetsci12111044 (PMC12656906; doi:10.3390/vetsci12111044)
Supplement: Supplementary file 1 [file vetsci-12-01044-s001.zip › Figure S1 MLST Clustering Analysis of Chinese Isolates of Mycoplasmosis bovis.pdf]

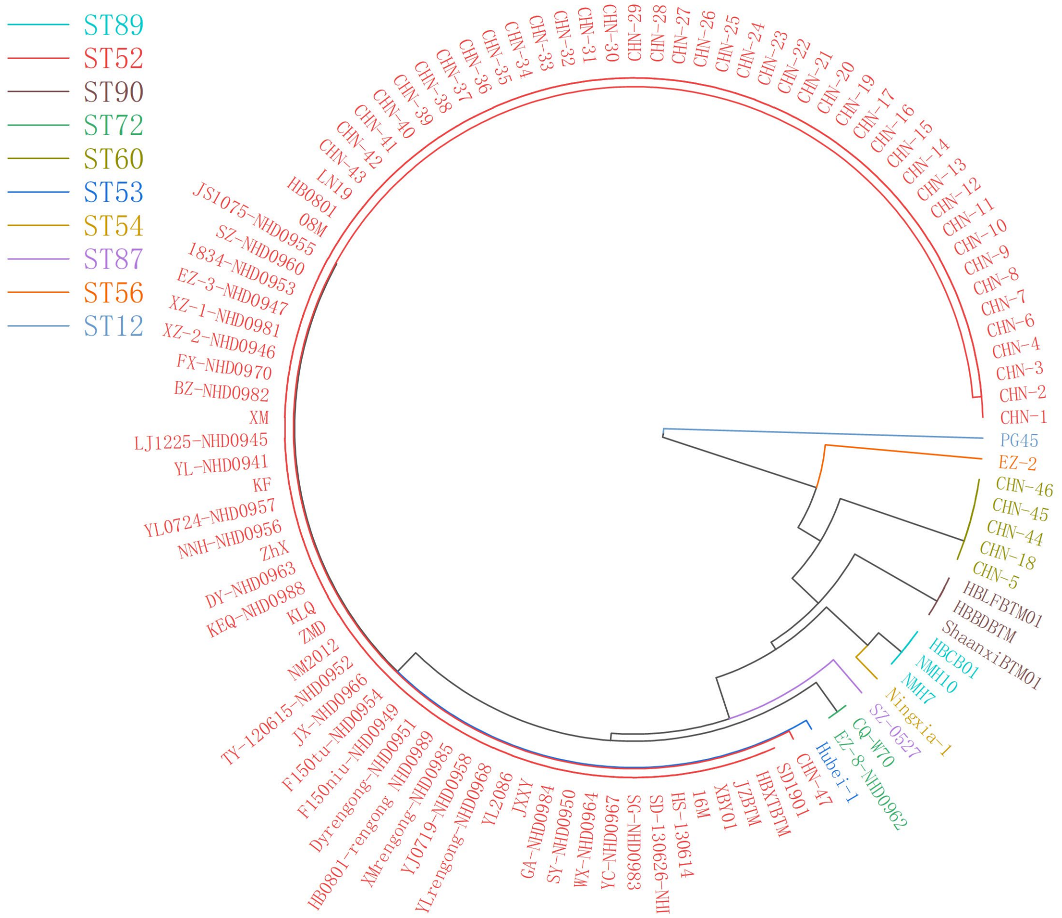

Figure S1. MLST Clustering Analysis of Chinese Isolates of Mycoplasmosis bovis. Note: CHN-1 to CHN-47, LN 19, and 16M were all clinical isolates in this study, and the others were obtained from the data of Chinese clinical isolates in the pubMLST database
