# Supplementary material for: Comprehensive Characterization of Mycoplasmosis bovis ST52 Strain 16M Reveals Its Pathogenicity and Potential Value in Vaccine Development
Source: Vet Sci. 2025 Nov 1;12(11):1044. doi: 10.3390/vetsci12111044 (PMC12656906; doi:10.3390/vetsci12111044)
Supplement: Supplementary file 1 [file vetsci-12-01044-s001.zip › Table S1 The scoring criteria for challenge experiments.pdf]

Table S1. The scoring criteria for challenge experiments

SN	Project	Index	Value	Scale standards
1	clinical symptom	Snot / cough	1	According to the clinical symptoms, only the highest score assigned, the highest is not more than 5 points.
		Respiratory symptoms and associated with conjunctivitis / arthritis/otitis	2	
		severe abdominal respiration	3	
		completely bedridden	4	
		Death	5	
2	shedding duration	Day 7	1	Statistics on the discharge at the corresponding time point, 1 point for each positive time, 5 points when calf death, and the highest is not more than 5 points.
		Day 14	1	
		Day 21	1	
		Day 28	1	
		Day 35	1	
3	Gross lesions	< 1%	1	Lung consolidation ratio = consolidation area / lung area, no consolidation to 0 points, the highest not more than 5 points.
		1% ~ 5%	2	
		5% ~ 10%	3	
		10% ~ 15%	4	
		> 15%	5	
4	Pathological change	Bronchiolar epithelium shedding and inflammatory cells infiltration	1	The pathological slides were taken from the same position of the diaphragm on one side of the consolidation. According to the degree of pathological change, only the highest score was assigned to the lesion, and the highest score was not more than 5 points.
		Alveolar interstitial cell hyperplasia	2	
		Forming a distinct sleeve structure	3	
		Alveolar fusion or collapse with inflammatory cell exudation	4	
		The majority of alveolar structures are completely destroyed and have lost their physiological function.	5	
