# Supplementary material for: Comprehensive Characterization of Mycoplasmosis bovis ST52 Strain 16M Reveals Its Pathogenicity and Potential Value in Vaccine Development
Source: Vet Sci. 2025 Nov 1;12(11):1044. doi: 10.3390/vetsci12111044 (PMC12656906; doi:10.3390/vetsci12111044)
Supplement: Supplementary file 1 [file vetsci-12-01044-s001.zip › Table S2 The result of the prediction for potential virulence genes in the genome of M. bovis 16M.pdf]

Table S2. The result of the prediction for potential virulence genes in the genome of *M. bovis 16M*

Database	Locus	Product	Gene	Protein length (aa)	Position
	SD1603_GM001446	fluoroquinolone resistance protein	gyrA	920	757680:760439:-
CARD	SD1603_GM001587	rifamycin resistance protein	rpoB	901	834969:837671:-
	SD1603_GM000846	aminocoumarin resistance protein	alaS	533	449127:450725:+
	SD1603_GM000655	Vpma like predicted lipoprotein	MAG2540	157	70834:71304:-
	SD1603_GM000184	Nitrogen fixation protein	nifS	235	77396:78100:+
	SD1603_GM000775	Alcohol dehydrogenase	adhT	250	90954:91703:+
	SD1603_GM000813	Alcohol dehydrogenase	adhT	210	93724:94353:+
	SD1603_GM001200	Resistance to a Wall teichoic acid (WTA)-binding bacteriophage	galU	151	99452:99904:+
	SD1603_GM000711	Iron utilization	secA	237	113163:113873:+
	SD1603_GM001429	DNA recombination and repair	recA	328	129416:130399:+
	SD1603_GM001916	Magnesium ion transporter	MgtB	319	172727:173683:+
	SD1603_GM000459	DNA gyrase (bacterial topoisomerase II)	GyrA	430	177552:178841:+
	SD1603_GM000460	DNA gyrase (bacterial topoisomerase II)	GyrA	430	181635:182924:+
	SD1603_GM001446	DNA gyrase (bacterial topoisomerase II)	GyrA	104	243165:243476:+
	SD1603_GM001876	Manganese Homeostasis	MntE	477	243480:244910:+
	SD1603_GM000350	Phosphate acetyltransferase	eutD	407	350160:351380:-
	SD1603_GM001305	Phosphate acetyltransferase	eutD	407	376965:378185:-
PHI	SD1603_GM000254	Predicted lipoprotein	MAG1050	238	410458:411171:+
	SD1603_GM000224	Pyruvate Dehyrogenase	pdhB	292	431316:432191:+
	SD1603_GM000177	Serine hydroxymethyl transferase	glyA	301	502783:503685:+
	SD1603_GM001419	transcription factor	GzOB009	67	582100:582300:+
	SD1603_GM001530	transcription factor	GzOB039	193	591877:592455:-
	SD1603_GM000358	Part of the pyruvate - tricarboxylic acid cycle node	pykF	275	641313:642137:+
	SD1603_GM000362	Part of the pyruvate - tricarboxylic acid cycle node	pykF	325	685616:686590:+
	SD1603_GM000152	Lipoate protein ligase	lplA1	267	742081:742881:-
	SD1603_GM001108	diadenylate cyclase	DacA	293	747064:747942:+
	SD1603_GM001499	Hypothetical protein	MGG_00383	920	757680:760439:-
	SD1603_GM001618	formyl methionyl transferase	FMT	277	787778:788608:-
	SD1603_GM001086	Methionine Sulfoxide Reductase	msrB	138	805166:805579:-
	SD1603_GM000948	Ribosomal RNA small subunit methyltransferase H	rsmH	176	856301:856828:-
	SD1603_GM000196	Fructose-bisphosphate aldolase	FbaA	359	984471:985547:-
	SD1603_GM000140	Glyceraldehyde-3-phosphate dehydrogenase	GAPDH	435	1002031:1003335:-

	SD1603_GM000018	Lipoate protein ligase A1	lplA1	149	9341:9787:-
	SD1603_GM000066	oppF oligopeptide ABC transporter, permease component	Capsule	196	33071:33658:-
	SD1603_GM000140	glyceraldehyde-3-phosphate dehydrogenase, type I	gapA	157	70834:71304:-
	SD1603_GM000152	Lipoate protein ligase A1	lplA1	235	77396:78100:+
	SD1603_GM000224	Pyruvate dehydrogenase E1 component, betasubunit	pdhB	237	113163:113873:+
	SD1603_GM000365	molecular chaperone DnaK	MOMP	371	185056:186168:+
	SD1603_GM001071	tuf Elongation factor Tu EF-Tu	EF-Tu	183	574202:574750:-
	SD1603_GM001072	tuf Elongation factor Tu EF-Tu	EF-Tu	201	574790:575392:-
	SD1603_GM001074	phosphopyruvate hydratase	-	252	575993:576748:+
VFDB	SD1603_GM001200	UTP--glucose-1-phosphate uridylyltransferase	gtaB	275	641313:642137:+
	SD1603_GM001548	msbA lipid A ABC exporter, fused ATPase and inner membrane subunits MsbA	LOS	332	813775:814770:-
	SD1603_GM001603	clpE ATP-dependent protease	ClpE	328	846538:847521:+
	SD1603_GM001604	clpE ATP-dependent protease	ClpE	312	847771:848706:+
	SD1603_GM001824	vamp Variable surface lipoprotein V VpmaVprecursor	Vpma	334	956894:957895:-
	SD1603_GM001828	vamp Variable surface lipoprotein V VpmaVprecursor	Vpma	334	959511:960512:-
	SD1603_GM001829	vamp Variable surface lipoprotein W VpmaWprecursor	Vpma	299	960671:961567:-
	SD1603_GM001916	Mg2+ transport	mgtB	435	1002031:1003335:-
