# Supplementary material for: Comprehensive Characterization of Mycoplasmosis bovis ST52 Strain 16M Reveals Its Pathogenicity and Potential Value in Vaccine Development
Source: Vet Sci. 2025 Nov 1;12(11):1044. doi: 10.3390/vetsci12111044 (PMC12656906; doi:10.3390/vetsci12111044)
Supplement: Supplementary file 1 [file vetsci-12-01044-s001.zip › Table S3 Genes predicted involving in putative lipoprotein and lipoprotein metabolic of M. bovis 16M.pdf]

Table S3. Genes predicted involving in putative lipoprotein and lipoprotein metabolic of *M. bovis 16M*

SN	Locus	Position	Product	Function	Secretory
1	SD1603_GM000401	{Chr1:207657:208133:+}	P48 major surface lipoprotein	certein function	NO
2	SD1603_GM001338	{Chr1:701465:702031:+}	membrane lipoprotein P81	certein function	NO
3	SD1603_GM000254	{Chr1:129416:130399:+}	putative lipoprotein	uncertein function	YES
4	SD1603_GM000254	{Chr1:129416:130399:+}	putative lipoprotein	uncertein function	YES
5	SD1603_GM000400	{Chr1:207021:207590:+}	putative lipoprotein	uncertein function	NO
6	SD1603_GM000403	{Chr1:208425:208985:+}	lipoprotein	uncertein function	NO
7	SD1603_GM000418	{Chr1:220711:222318:+}	lipoprotein	uncertein function	NO
8	SD1603_GM000475	{Chr1:252392:253402:-}	Hypothetical protein, predicted lipoprotein	uncertein function	NO
9	SD1603_GM000477	{Chr1:253719:254774:-}	Hypothetical protein, predicted lipoprotein	uncertein function	NO
10	SD1603_GM000499	{Chr1:263871:264263:+}	putative lipoprotein	uncertein function	YES
11	SD1603_GM000629	{Chr1:334465:334779:+}	lipoprotein	uncertein function	NO
12	SD1603_GM000654	{Chr1:348348:349700:-}	PKD repeat putative lipoprotein	uncertein function	NO
13	SD1603_GM000663	{Chr1:354973:355791:-}	putative lipoprotein	uncertein function	NO
14	SD1603_GM000752	{Chr1:396289:396828:-}	Lipoprotein, putative acid phosphatase	uncertein function	NO
15	SD1603_GM000811	{Chr1:430748:430939:+}	lipoprotein	uncertein function	NO
16	SD1603_GM000824	{Chr1:437147:437293:-}	lipoprotein	uncertein function	NO
17	SD1603_GM000885	{Chr1:467636:468415:+}	putative membrane lipoprotein (ICEB-1 encoded)	uncertein function	NO
18	SD1603_GM001027	{Chr1:545610:545993:-}	putative membrane lipoprotein	uncertein function	NO
19	SD1603_GM001028	{Chr1:546293:547351:+}	Hypothetical protein, predicted lipoprotein	uncertein function	YES

20	SD1603_GM001054	{Chr1:560076:560384:+}	lipoprotein	uncertein function	NO
21	SD1603_GM001055	{Chr1:560847:561320:+}	Hypothetical protein, predicted lipoprotein	uncertein function	NO
22	SD1603_GM001058	{Chr1:566483:566608:-}	lipoprotein	uncertein function	NO
23	SD1603_GM001059	{Chr1:566878:567024:-}	lipoprotein	uncertein function	NO
24	SD1603_GM001138	{Chr1:605175:605552:-}	lipoprotein	uncertein function	YES
25	SD1603_GM001188	{Chr1:632766:633683:-}	putative surface prolipoprotein	uncertein function	YES
26	SD1603_GM001483	{Chr1:778099:778713:+}	Hypothetical protein, predicted lipoprotein	uncertein function	NO
27	SD1603_GM001543	{Chr1:810728:811498:+}	putative lipoprotein	uncertein function	YES
28	SD1603_GM001593	{Chr1:840910:841662:+}	lipoprotein	uncertein function	NO
29	SD1603_GM001836	{Chr1:965639:966130:+}	putative lipoprotein	uncertein function	NO
30	SD1603_GM000630	{Chr1:334837:335031:+}	variable surface lipoprotein	variable surface lipoprotein	NO
31	SD1603_GM000865	{Chr1:459045:459491:+}	putative variable surface prolipoprotein	variable surface lipoprotein	NO
32	SD1603_GM000995	{Chr1:528880:529683:+}	variable surface lipoprotein Y	variable surface lipoprotein	YES
33	SD1603_GM001784	{Chr1:940373:940831:+}	variable surface lipoprotein, VspI	variable surface lipoprotein	YES
34	SD1603_GM001818	{Chr1:953563:954195:-}	chimeric variable surface lipoprotein	variable surface lipoprotein	YES
35	SD1603_GM001821	{Chr1:955965:956375:+}	variable surface lipoprotein Vsp422-1	variable surface lipoprotein	YES

36	SD1603_GM001824	{Chr1:956894:957895:-}	variable surface lipoprotein, VspL	variable surface lipoprotein	YES
37	SD1603_GM001825	{Chr1:958596:958958:+}	variable surface lipoprotein Vsp422-6	variable surface lipoprotein	YES
38	SD1603_GM001828	{Chr1:959511:960512:-}	variable surface lipoprotein, VspL	variable surface lipoprotein	YES
39	SD1603_GM001829	{Chr1:960671:961567:-}	variable surface lipoprotein VspHB0801-4	variable surface lipoprotein	YES
40	SD1603_GM000655	{Chr1:350160:351380:-}	Vpma-like lipoprotein	Vpma-like lipoprotein	YES
41	SD1603_GM000655	{Chr1:350160:351380:-}	Vpma-like, lipoprotein	Vpma-like lipoprotein	YES
42	SD1603_GM000216	{Chr1:109581:109760:+}	Prolipoprotein diacylglyceryl transferase	lipoprotein metabolic	NO
43	SD1603_GM000849	{Chr1:451610:451900:+}	Conserved hypothetical protein	lipoprotein metabolic	NO
44	SD1603_GM001918	{Chr1:1004371:1005459:-}	ABC-type lipoprotein export system, ATPase component	lipoprotein metabolic	NO
