# Supplementary figures and images for: Comprehensive Characterization of Mycoplasmosis bovis ST52 Strain 16M Reveals Its Pathogenicity and Potential Value in Vaccine Development
Source: Vet Sci. 2025 Nov 1;12(11):1044. doi: 10.3390/vetsci12111044 (PMC12656906; doi:10.3390/vetsci12111044)

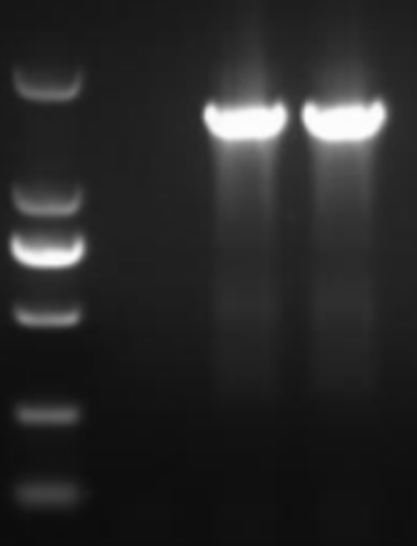

Supplement: Supplementary file 1 [file vetsci-12-01044-s001.zip › Figure S2 Original image of Figure 1b.tif]
